# 3D-Printing for Critical Sized Bone Defects: Current Concepts and Future Directions

**DOI:** 10.3390/bioengineering9110680

**Published:** 2022-11-11

**Authors:** Cory K. Mayfield, Mina Ayad, Elizabeth Lechtholz-Zey, Yong Chen, Jay R. Lieberman

**Affiliations:** 1 Department of Orthopaedic Surgery, Keck School of Medicine of USC, Los Angeles, CA 90033, USA; 2 Department of Aerospace and Mechanical Engineering, Viterbi School of Engineering, University of Southern California, Los Angleles, CA 90089, USA

**Keywords:** 3D printing, segmental bone defect, orthopaedic surgery, bone healing, gene therapy

## Abstract

The management and definitive treatment of segmental bone defects in the setting of acute trauma, fracture non-union, revision joint arthroplasty, and tumor surgery are challenging clinical problems with no consistently satisfactory solution. Orthopaedic surgeons are developing novel strategies to treat these problems, including three-dimensional (3D) printing combined with growth factors and/or cells. This article reviews the current strategies for management of segmental bone loss in orthopaedic surgery, including graft selection, bone graft substitutes, and operative techniques. Furthermore, we highlight 3D printing as a technology that may serve a major role in the management of segmental defects. The optimization of a 3D-printed scaffold design through printing technique, material selection, and scaffold geometry, as well as biologic additives to enhance bone regeneration and incorporation could change the treatment paradigm for these difficult bone repair problems.

## 1. Introduction

Segmental and critical sized bone defects in orthopaedic surgery remain a challenging clinical scenario for both the surgeon and patient. Bone loss within orthopaedics comprises a continuum of injuries. Present surgical techniques enable the successful treatment of small bone defects (<1 cm) with a well-preserved soft tissue envelope using a variety of options including autologous bone grafting, bone marrow aspirate injection, and allograft options such as demineralized bone matrix. However, the treatment of larger segmental or critical sized bone defects involve a number of factors including the presence of bone comminution and a compromised soft tissue envelope (i.e., the status of the periosteum, vascular supply, adjacent musculature, fascia, and cutaneous tissue) [1]. Whereas a segmental defect refers to an absent section of bone within a limb segment, a critical sized defect refers to a defect that is unable to heal without additional intervention, and most segmental defects are considered to be a “critical size” [2,3]. The exact dimensions and size of a critical size defect vary based on the location within the body as well as patient factors, but have been previously described as bone voids 2 to 2.5 times the diameter of the affected bone or circumferential bone loss >50% for non-segmental defects [4,5,6,7]. These types of bone defects occur in a number of settings, including acute trauma, fracture nonunion, osteomyelitis, neoplasia, spine pseudarthrosis, and revision total joint arthroplasty [3]. The associated healthcare costs for segmental bone defects are estimated as high as $300,000 per case, with significant associated patient morbidity [8].

The healing of large bone defects is a multifaceted process that requires several essential elements, including: an osteoconductive scaffold or matrix; responsive osteogenic cells; bioactive growth factors; and an adequate blood supply. Osteoconductive materials are those which provide a structure and microenvironment that promote chemotaxis, deposition of host bone, and ingrowth of host capillaries and perivascular tissue [9]. Osteoinductive materials are those which deliver growth factors—namely bone morphogenetic proteins (BMPs), fibroblast growth factor (FGF), vascular endothelial growth factor (VEGF), and platelet derived growth factor (PDGF)—that drive differentiation of primitive pluripotent stem cells into bone-forming cell lineages [10]. Osteogenesis refers to the process by which new bone is created and deposited by the host. For a graft to possess osteogenic properties, it must be both osteoconductive and osteoinductive, as well as deliver mesenchymal stem cells (MSCs), osteoblasts, and osteocytes [9]. Osseointegration is the process by which new host bone is incorporated into previously existing bone through substitution and remodeling [9]. Given these considerations, the ideal treatment strategy must provide all these elements in a cost-effective manner while minimizing patient morbidity.

While numerous treatment strategies and bone substitutes are utilized to address bone loss in clinical practice, there remains an unmet clinical need in the treatment of segmental defects, with no “silver-bullet” that provides an osteoconductive and osteoinductive environment while eliminating donor site and patient morbidity. Three-dimensional (3D) printing of scaffolds that can facilitate the delivery of cells or growth factors has emerged as a potential technology to address these limitations. 3D printing is an attractive technology for segmental bone defects given its ability to customize the scaffold to the size and shape of the bone defect, and the ability to create a scaffold that stimulates and enhances the bone repair process. Ideally, clinicians would utilize a computed tomography (CT) scan of the patient’s limb to generate a custom 3D-printed scaffold to fit the desired defect dimensions and further augment the scaffold with growth factors, stem cells, or genetically manipulated stem cells. Designing the optimal scaffold is dependent on a variety of factors including intrinsic osteoconductive properties, ability to deliver osteoinductive growth factors or osteogenic cells, complete incorporation into host bone, and eventual resorption at a rate that allows for appropriate bone remodeling.

The purpose of this review is to discuss the current treatment options for the management of segmental bone defects along with their associated benefits and limitations. Furthermore, we aim to highlight 3D-printing technology supplemented with growth factors or cells as a promising treatment option in the management of bone loss in orthopaedic surgery. We discuss the techniques, properties, rationale, and material choices of 3D printing, as well as future directions for application in clinical practice.

## 2. Current Strategies for Segmental Bone Loss

### 2.1. Current Surgical Techniques for Addressing Bone Loss

#### 2.1.1. Induced Membrane Technique

**Figure 1:**
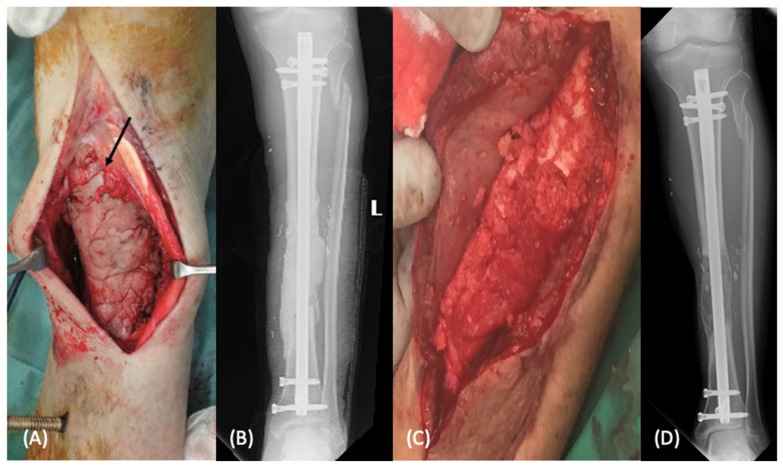
Masquelet technique for diaphyseal tibial defect demonstrated in intraoperative photos of the antibiotic spacer placement (**A**) with post-operative radiographs (**B**). The patient underwent subsequent second stage autologous bone grafting into induced membrane (**C**) with post-operative radiographs seen in image (**D**).

Autologous bone grafting has demonstrated success for defects less than 5 cm. However, high failure rates in defects larger than 5 cm led to the development of the induced membrane technique by Masquelet and Begue [11]. This two-stage procedure initially involves the implantation of a polymethyl methacrylate (PMMA) cement spacer at the site of the bone defect for approximately 6–8 weeks (Figure 1). This PMMA cement spacer induces the formation of a thick, vascularized membrane which can then be carefully incised to remove the spacer and fill with bone graft [11]. The Masquelet technique has several proposed benefits in the treatment of segmental defects, the first being creation of a separate compartment at the defect site to prevent adjacent soft tissue interposition and allow retention of autologous bone graft following the second stage procedure. Second, the PMMA cement spacer allows for maintenance of the defect site and length of fracture site for future grafting. Lastly, the creation of an induced pseudoperiosteum that is well vascularized, rich in type I collagen, and secretes growth factors (including BMP-2, VEGF, TGF-β1, and IL-6) promotes graft consolidation and remodeling through cell proliferation and osteoblastic differentiation [12,13]. This technique is particularly advantageous in the setting of infection associated with acute bone loss given that antibiotics can be loaded into the PMMA cement to decrease bacterial counts at the defect site [14]. Clinically, the induced membrane technique has demonstrated reliable results in the management of segmental defect with the original series by Masquelet and Begue demonstrating an 89% union rate [11]. While no prospective randomized trials investigating the induced membrane technique have been performed, a recent meta-analysis of 1386 cases treated with the induced membrane technique demonstrated a union rate of 82.3% and a mean time to union of 6.6 months, with a protective effect towards additional procedures seen with antibiotic-loaded PMMA [15]. The limitations of the induced membrane technique include the requirement of multiple surgical interventions, risk of deep space infection, graft resorption, and persistent non-union or infection [15].

#### 2.1.2. Distraction Osteogenesis

**Figure 2:**
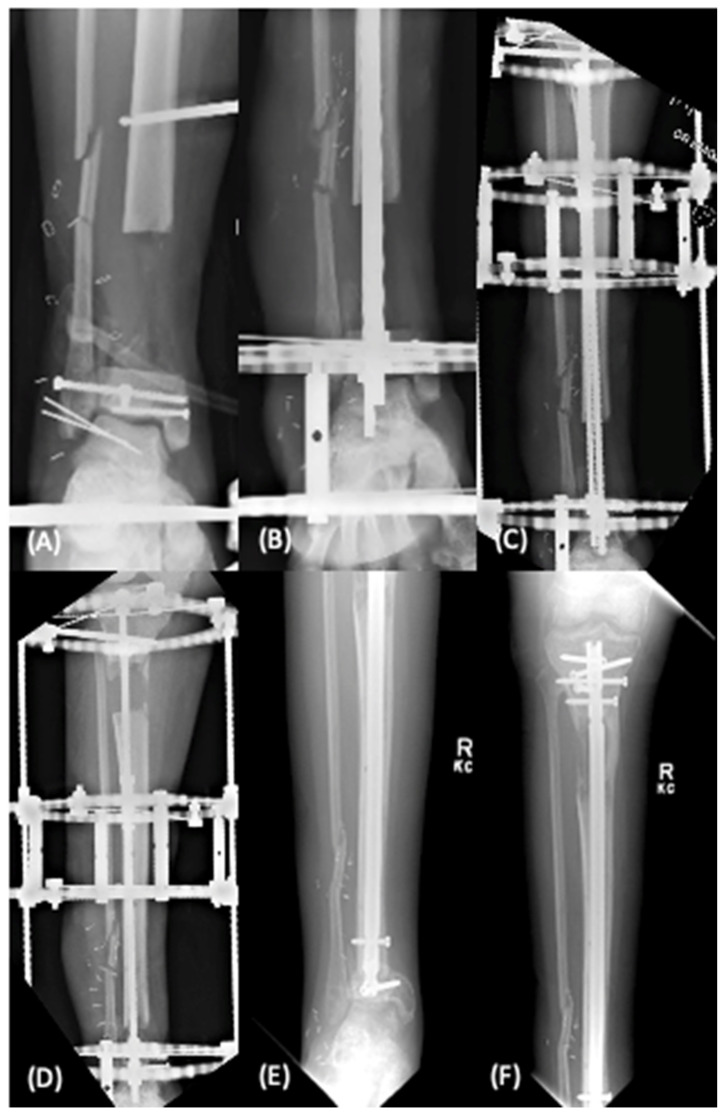
Distraction osteogenesis for the management of distal tibial metadiaphyseal defect (arrow). Initially treated with debridement and temporary hybrid fixation (**A**). Subsequently underwent first stage procedure involving intramedullary nail and ringed external fixator placement and proximal tibia corticotomy (**B**,**C**). Midpoint follow up demonstrates proximal to distal transport (**D**). After completion of transport, subsequent docking, and removal of ringed external fixator with proximal deposition of bone (**E**,**F**).

Originally described by Ilizarov, distraction osteogenesis involves the creation and transport of a segment of bone in a controlled fashion with the use of an external fixator or intramedullary transport nail [16]. Initially this technique used circular fine-wire frames to treat nonunions; however, it has expanded to include modern spatial external fixator frames and intramedullary transport nails. Through bone transport, controlled mechanical strain is applied to the transport bone segment to induce both intramembranous and endochondral ossification [17]. The procedure involves a metaphyseal corticotomy followed by a 5–10 day latent period prior to distraction at a rate of 1 mm per day (Figure 2). Once the bone transport segment reaches the distal aspect of the fracture, the docking phase begins as the distal segment is compressed until fracture union is achieved, usually lasting twice as long as the transport phase [18]. If incomplete callus formation is seen during the docking phase, a secondary autograft procedure is performed in conjunction with compression internal fixation to induce bone healing.

The primary advantage of distraction osteogenesis in treating segmental bone defects is the ability to bear weight during transport. The main disadvantage of this technique is the duration of time required for reconstruction (average of 6–12 month), which can place significant psychological burden on the patient [19]. Other disadvantages of this procedure include prolonged external fixation with the risk of pin-tract infection, risk of fracture of the regenerated segment, joint stiffness, limb length discrepancy, neurovascular complications, and need for amputation [3,19]. In a systematic review by Papakostidis involving 37 studies with 898 patients, the overall union rate was 94%; however, the authors highlighted the rates of associated complications. In their pooled analysis, the refracture rate was 5% with increased risk of fracture in defects >8 cm. Additionally, they noted a 2.2% incidence of neurovascular complications and a 2.9% incidence of amputation, over half of which (1.6%) were voluntary [19].

#### 2.1.3. Vascularized Bone Grafting

**Figure 3:**
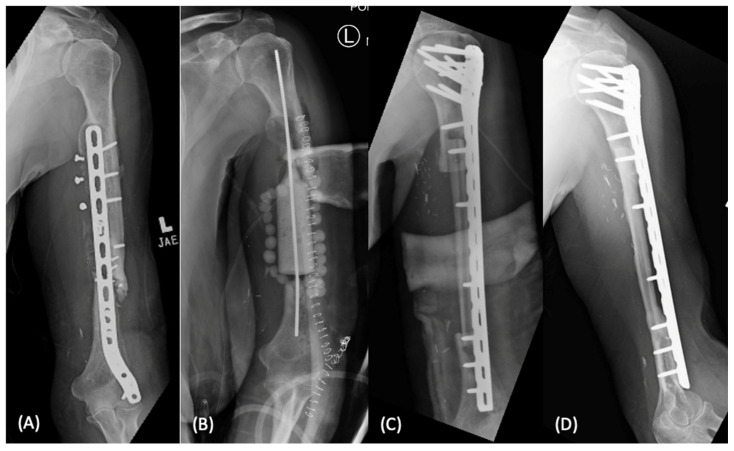
Management of infected humeral shaft non-union with free vascularized fibula transfer (arrow) (**A**). Initially treated with removal of hardware, debridement, and temporary antibiotic cement spacer (**B**). Subsequently, the patient underwent vascularized free fibula transfer and revision open reduction internal fixation (**C**), with excellent graft incorporation at one-year follow up (**D**).

Given the evolution of microsurgical techniques, vascularized bone grafting allows for immediate transfer of graft with associated vascular supply to provide both mechanical stability and bony architecture to the defect site. Vascularized bone grafting is a technically demanding procedure that requires advanced microsurgical expertise to successfully complete the anastomosis at the recipient site. Numerous vascularized graft options have been demonstrated including the fibula, medial femoral condyle, and distal radius perforators, however the fibula is considered ideal for long bone diaphyseal defects (Figure 3). This is due to the long cylindrical shape, mechanical strength, predictable vascular pedicle, and potential for hypertrophy once transferred [20]. The primary advantage of the vascularized bone graft is the preservation of the blood supply, which allows for maintenance of osteogenic potential and primary or secondary bone healing [21]. This results in less graft resorption and decreased risk of mechanical failure as compared to nonvascularized bone graft [9]. Clinically, excellent union rates of 77–100% have been noted across multiple areas of orthopaedics, including long bone diaphyseal defects, upper extremity surgery, and oncologic reconstructions [22,23,24,25]. The disadvantages of vascularized bone grafting include a prolonged period of partial weight bearing to allow graft hypertrophy, risk of graft fracture due to premature weight bearing, anastomotic complications, and donor site morbidity [26].

#### 2.1.4. Growth Factor Augmentation with BMP

Bone Morphogenetic Protein (BMP), a member of the TGF-β superfamily, is the most potent osteoinductive graft agent that is commercially available today [10]. rhBMP-2 (INFUSE^®^ Bone Graft, Medtronic Sofamor Danek USA Inc., Memphis, TN, USA) has FDA approval for usage in acute open tibia fractures and anterior lumbar interbody fusion (ALIF) [27,28]. Although BMP-2 is osteoinductive, the clinical results have been mixed. Large doses of BMP are required to induce an osteoinductive biologic response in humans, and these doses are associated with heterotopic ossification and soft tissue swelling [29]. The development of more efficient protein delivery systems may allow for a dose reduction or more sustained protein release that can enhance the osteoinductive activity of BMP.

### 2.2. Current Scaffolds

#### 2.2.1. Autograft

The current “gold-standard” bone graft material is autologous autograft, with nearly 500,000 bone grafting procedures performed annually in the United States [30,31]. Autografting involves utilizing bone that is harvested from the host and can be obtained from number of sites including the anterior or posterior iliac crest, intramedullary femoral or tibial canal (reamer irrigator aspirator), distal radius, medial femoral condyle, proximal tibia, or calcaneus [32,33,34,35,36]. Autologous bone has numerous advantages in that it is readily available; has osteoconductive, osteoinductive, and osteogenic properties; and avoids any risk of immunogenicity or disease transmission [31,37,38,39,40]. However, the donor site morbidity and complications associated with autologous graft harvesting are well-known. The morbidity of a second surgical site for donor harvest with reported chronic pain, increased operative time, increased blood loss, risk of neurovascular injury, fracture, dysthesia, infection, and cosmetic deformity have been well-reported [41,42,43]. In addition, there is only a limited amount of autogenous bone available from every patient.

#### 2.2.2. Allograft

Given the continued rising need for bone graft in the setting of trauma as well as spinal fusion and revision total joint arthroplasty, there has been interest in using allograft as an available alternative to autograft. Allogenic bone grafting involves using tissue that is harvested from one individual and transplanted into another [44]. The primary advantage to allograft usage is the immediate availability with numerous size options available. Bone allograft is available as cortical or cancellous graft, or as demineralized bone matrix (DBM) [45]. These grafts are available through a number of processing methods, including fresh, fresh-frozen, freeze-dried, demineralized, and gamma irradiated [46]. Fresh-frozen cortical allografts retain osteoconductive properties but are not osteoinductive. This graft type is particularly useful in the management of articular defects, but is associated with >50% long-term failure rates [47]. One important risk of allografts, particularly fresh-frozen, is the risk of potential transmission of viruses including HIV, hepatitis B, and hepatitis C, as well as bacterial infection transmission [48]. Ethanol prepared, ethylene oxide treated, or freeze-dried graft that undergoes gamma irrigation reduces this risk of disease transmission, however, these processes weaken the mechanical properties of the graft, remove osteogenic components, and limit their osteoinductivity [49].

Conversely, DBM is allogenic bone that has been processed to micron size and demineralized using hydrochloric acid [50]. The commercially available DBMs have different properties, but they are osteoconductive but not osteoinductive. Allograft DBMs contain type I collagen, non-collagenous proteins, and bone-inducing growth factors, including BMP, transforming growth factor (TGF), and FGF; they are not osteoinductive in humans. Clinically, DBM has been shown to be ineffective when used in isolation to treat nonunion or segmental defects, and is used most commonly in combination with cortical or cancellous allograft as a means to expand the volume of graft that can be delivered [51,52]. Newer products such as Trinity Evolution/ELITE (OrthoFix Medical Inc., Lewisville, TX, USA) and Osteocel Plus (NuVasive Inc., San Diego, CA, USA) contain allograft bone materials in addition to viable mesenchymal stem cells, osteoblasts, and osteocytes in an effort to add osteogenic and osteoinductive properties to allograft. However, these products are limited in their applications most significantly by cost and limited long-term clinical follow-up [53,54].

#### 2.2.3. Synthetic Bone Substitutes

Synthetic alternatives to autograft and allograft have been developed as bone graft substitutes. These synthetic substitutes come in a variety of forms including pellets, powders, putty, solid blocks, and injectable pastes. The four main classes of synthetic bone substitutes available include calcium sulfate, calcium phosphate, tricalcium phosphate, and hydroxyapatite. Synthetic substitutes do not have osteogenic or osteoinductive properties, but instead obviate the risks and costs associated with autograft harvest as well as the potential for disease transmission in allografts while providing osteoconductive scaffolds [55]. Due to their strictly osteoconductive nature, these substitutes are rarely used in isolation for fracture nonunion or segmental bone defects. Instead, because of their high compressive strength in filling bone voids, these synthetic materials are particularly useful for providing structural support. In addition, these materials can deliver supratherapeutic local concentrations of antibiotics in revision joint arthroplasty and infected nonunions. High quality evidence in the orthopaedic literature supporting the use of synthetic bone graft substitutes remains limited. A systematic review by Kurien et al. [56] investigating bone graft substitutes in the setting of trauma, nonunion, and revision total hip arthroplasty noted that Level I evidence supporting the use of bone graft substitutes was present for only four of the commercially available products. These osteoconductive agents do not have the biologic potential to be used alone to treat segmental bone defects.

## 3. Future Directions for Addressing Bone Loss

### 3.1. Multiple Stage “Bioreactor”

As different techniques for bone tissue engineering emerge, a common concern is immunoreactivity and suboptimal microenvironments for both angiogenesis and osteogenesis. Proper vascularization of bone grafts is essential for infection resistance, as well as osteoconduction and osseointegration of the scaffold into the surrounding bone [57]. To address these obstacles, a novel approach involves the in vivo implantation of a scaffold into an ectopic site such as skin or muscle, termed an in vivo bioreactor (IVB), with subsequent transfer of the newly vascularized scaffold into the bony site of interest [58,59,60,61,62]. To date, many preclinical studies have demonstrated robust bone healing responses using the IVB method [63,64,65,66]. The majority of IVB use in humans involves grafting periosteum along with its vascular supply to the site of interest [67,68,69,70], but actual prefabrication techniques involving ectopic maturation of a scaffold with subsequent transfer to the site of interest are rare in clinical studies. To date, clinical reports utilizing IVB have focused primarily on facial bone and mandibular defects [60,71,72]. This strategy has clinical potential but the appropriate scaffolds, growth factors, and cells must be identified for various clinical scenarios.

### 3.2. 3D Printing—Current Techniques

3D printing is a promising emerging technology for the management of bone loss in orthopaedics given the ability to modify and optimize the size and shape of the scaffold using preoperative CT for each patient. The “ideal” scaffold must address many factors including containing inherent osteoconductive properties, mechanical stability, remodeling potential, favorable material resorption profile, allowing vascular ingrowth, and delivery of osteoinductive factors or cells. Through modulation of printing technique, material selection, pore size and geometry, scaffold surface topography, and augmentation with cells or growth factors, providers will be able to individualize the treatment of segmental bone defects and tailor 3D-printed scaffold design and augmentation to the specific needs of the patient.

#### 3.2.1. Fused Deposition Modeling

**Figure 4:**
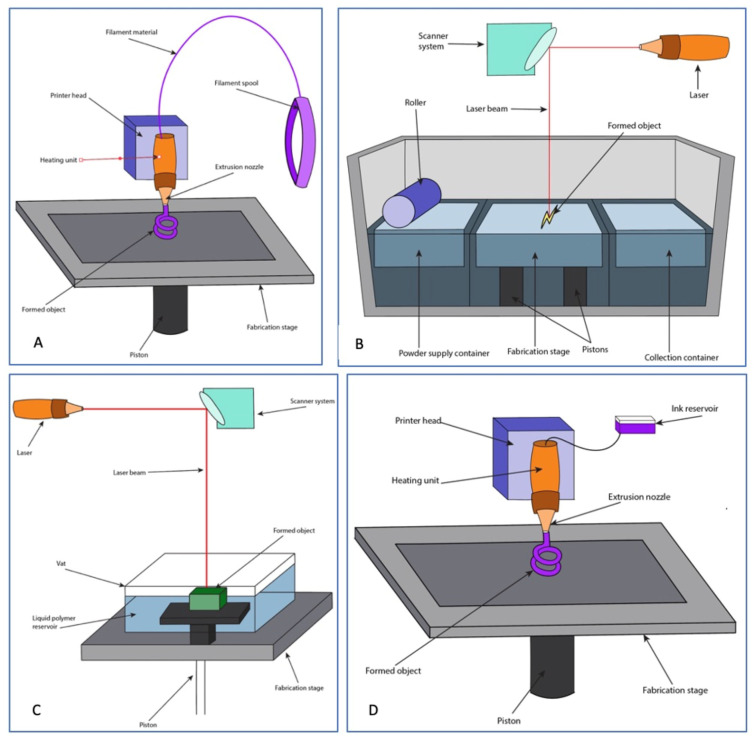
Schematic representations of 3-D printing techniques. (**A**) Fused deposition modeling. (**B**) Selective laser sintering. (**C**) Stereolithography. (**D**) Robotic material extrusion (robocasting).

Fused deposition modeling (FDM) is a popular method of additive 3D printing based on the precise extrusion of plastic polymers from a heated nozzle onto a cooler substrate, allowing for rapid solidification of the extruded filament (Figure 4A). This technique is frequently used for bone tissue engineering applications due to its reliability and precision, as well as the ease of creating biocompatible scaffolds based on CT-guided microspecifications of bone defect dimensions (Table 1) [73]. FDM scaffolds can be developed from a variety of materials, including polylactic acid, hydroxyapatite, ß-tricalcium phosphate, and bioactive glass [74,75]. As bone tissue engineering strategies become more complex, scaffolds are often supplemented with stem cells or growth factors to improve the osteogenic potential of the implanted construct. Due to the high temperatures required in extrusion during FDM, these biological materials are typically added to the scaffolds after they have sufficiently cooled [76]. 

Several studies have reported success in animal models with in vivo bone healing using FDM-generated scaffolds both with [86,87] and without [88,89] associated stem cells. Eichholz et al. demonstrated that human MSC-seeded FDM scaffolds used to treat critically sized murine femoral defects resulted in both robust bone formation and neovascularization throughout the pores of the scaffold [86]. Nulty et al. supplemented 3D-printed FDM polycaprolactone scaffolds with fibrin bioink containing human umbilical vein endothelial cells and human bone marrow stem cells and implanted these constructs into rat femoral defects. MicroCT and histological analysis revealed ongoing new bone formation and angiogenesis with confirmation by colony-forming unit assay [87]. These recent discoveries provide valuable insight into the feasibility of both cell-laden and cell-free FDM scaffolds in a clinical context for human bone tissue engineering.

#### 3.2.2. Selective Laser Sintering

Selective laser sintering (SLS) is a 3D printing technique that involves the use of a CO_2_ laser that sinters sequential layers of raw material in powders to create a 3D construct (Figure 4B) [76]. The advantages of using SLS in bone tissue engineering are that it allows for the generation of smaller scaffolds with more precise specifications, although the density of the construct is often sacrificed (Table 1) [81]. For this reason, several in vitro studies have investigated the feasibility of promoting the liquid phase of this technique in an effort to homogenize the microstructure formation during cooling and have shown preservation of biocompatibility through in vitro assays [82,83]. Several studies have demonstrated success with using cell-seeded SLS-generated scaffolds to heal segmental bone defects [90,91]. Kanczler et al. cultured human fetal femur-derived cells on SLS-generated polylactic acid scaffolds and implanted them both subcutaneously and into segmental murine femoral defects. Both in vitro and in vivo assays confirmed cell proliferation and ingrowth into the scaffold, as well as substantial callus formation and bone bridging as seen radiographically and histologically [90]. Xia et al. used nano-hydroxyapatite/poly-ε-caprolactone scaffolds seeded with human bone marrow stromal cells to heal rabbit segmental femoral defects and demonstrated superior cell proliferation and osteogenic potential both in vitro and in vivo as compared to the reference PCL-only scaffold [91]. Despite these advances, further investigation into how prolongation of the liquid phase affects both mechanical strength and cell proliferation in vivo will be pertinent to creating scaffolds for larger animal models.

#### 3.2.3. Stereolithography (Vat Photopolymerization)

Stereolithography apparatus (SLA) is an ultraviolet (UV) light-based 3D printing method that involves layered curing of a photopolymer resin, a process that allows for the generation of precise, high-resolution constructs (Figure 4C) [75]. Bio-ceramic parts can also be fabricated by SLA using a mixture of photopolymer resin and ceramic particles, followed by a debinding and sintering process [92,93]. In vitro assessments of SLA biocompatibility indicate that these scaffolds provide an environment conducive to osteogenic differentiation and proliferation of several different cell types, including human periosteal-derived stem cells [77], human mesenchymal stem cells [78,79,80], and human embryonic stem cells (Table 1) [94]. In vivo, SLA-generated scaffolds have shown great success in promoting de novo bone formation, angiogenesis, and osseointegration of the scaffold into murine and rabbit calvarial defects [78,95,96]. Using a combination of poly(propylene fumarate) and diethyl fumarate (DEF) as starting materials for the SLA scaffold, Lee et al. demonstrated that the addition of microspheres containing BMP-2 into the scaffold resulted in effective bone formation in murine calvarial defects [97]. Wei et al. utilized a canine model to demonstrate that BMSC-seeded scaffolds made from a calcium phosphate/monoalcohol ethoxylate phosphate slurry resulted in excellent angiogenesis and ectopic bone formation when implanted into dorsal muscles [98]. Current investigations are focused on bone formation ectopically or in calvarial defects, but additional investigation into the role of SLA-generated scaffolds in repairing defects in weight-bearing bones is necessary to translate to orthopaedics [97,99].

#### 3.2.4. Robotic Material Extrusion (Robocasting)

Robotic material extrusion, also known as robocasting, is a subtype of additive 3D printing whereby a high-viscosity paste bioink is dispensed by a printer’s nozzle in a layered fashion to create a 3D structure (Table 1) [75]. Because this method employs relatively low temperatures, it can be used to print bioactive materials including cells and modulate scaffold microarchitecture [84,85]. Assessments of the mechanical and compressive properties of porous scaffolds containing various organic and inorganic components have demonstrated excellent retention of mechanical strength after in vitro exposure to simulated body fluids [100,101]. Monfared et al. created composite BG/β-TCP scaffolds seeded with human adipose-derived MSCs and found that the high degree of precision afforded by robocasting resulted in exceptional mechanical strength. Additionally, scanning electron microscopy revealed uniform cell distribution both within and on the surface of the scaffold, indicating that the specific surface architecture was conducive for cell health and proliferation [102]. Kazemi et al. investigated a strontium-substituted β-TCP and bioactive glass scaffold seeded with rabbit BMSCs and revealed robust osteoinductive properties and osseointegration into the defect, as evidenced by the lack of a clear border between the remaining scaffold material and new bone [103]. Additional in vivo studies have investigated the role of scaffolds as treatments for rabbit femoral condyle defects [104,105], as well as 6 mm rabbit and rat femoral shaft defects [106,107,108]. The results of these studies indicate that scaffolds generated by robocasting have sufficient biomechanical strength and cell adhesion/proliferation properties to support bone regeneration in weight-bearing limbs but the efficacy of this technique needs to be evaluated in clinically relevant animal models.

#### 3.2.5. Bioprinting

##### Inkjet Bioprinting (Thermal and Piezoelectric)

**Figure 5:**
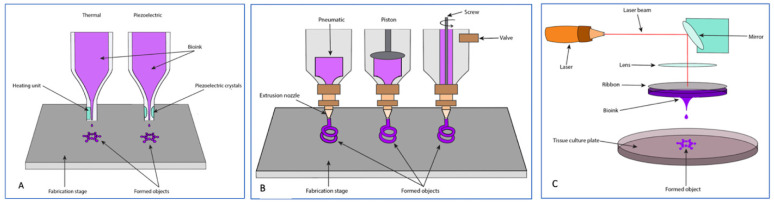
(**A**) Thermal and piezoelectric inkjet printing. (**B**) Ink extrusion bioprinting. (**C**) Laser-assisted bioprinting.

Inkjet bioprinting involves dripping a low-viscosity ink onto a substrate based on a computer program to create a 3D construct, and has been applied in multiple tissue regeneration contexts [76]. The two main subtypes of inkjet printing for tissue engineering are thermal and piezoelectric, both of which allow for the rapid creation of scaffolds with highly resolute microarchitecture [84]. Thermal inkjet printing involves using heat to vaporize the ink in the printhead, creating a bubble that expands until the ink is propelled through the nozzle and onto the substrate below. In piezoelectric inkjet printing, an electric current is applied to a piezoelectric crystal within the printhead, generating pressure that dispenses the ink (Figure 5A) [75]. Thermal inkjet printing has been explored as a method of simultaneously printing cells with various starting materials to improve cellular distribution and integration into the scaffold. Using this strategy, cell survivability has been reported between 86% and 98% with excellent osteogenic differentiation and collagen deposition [109,110,111]. Barui et al. fabricated a medical-grade titanium scaffold seeded with mouse fibroblasts and pre-osteoblasts and demonstrated excellent cell adhesion and viability, as well as mechanical strength comparable to that of cortical bone, indicating potential for use in in vivo applications [112].

A commonly cited concern with thermal inkjet printing is the difficulty with standardizing droplet size, as well as limitations with starting materials due to the low viscosity necessary for proper expulsion of the ink droplet [113,114]. Piezoelectric inkjet printing has the potential to overcome these obstacles, but has not been well studied in bone tissue engineering. However, this inkjet printing technique has shown promise in maintaining cell viability in vitro models and its use may be broadened to bone tissue engineering for in vivo study as 3D printing technology evolves [115,116,117].

##### Extrusion Bioprinting

Extrusion bioprinting, also referred to as direct ink writing (DIW), involves the use of pneumatic air pressure or mechanical systems such as pistons, screws, and valves to continuously disperse multiple different bioinks simultaneously, allowing for fabrication of scaffolds that emulate highly complex tissues (Figure 5) [75,118]. Some disadvantages of this technique include cellular exposure to high mechanical and shearing stresses during the extrusion process, as well as limited resolution of the final construct (Table 2) [114]. However, multiple studies report high levels of cell viability with cell-laden bioinks, as well as robust osteogenic and chondrogenic differentiation [119,120,121].

**Figure 6:**
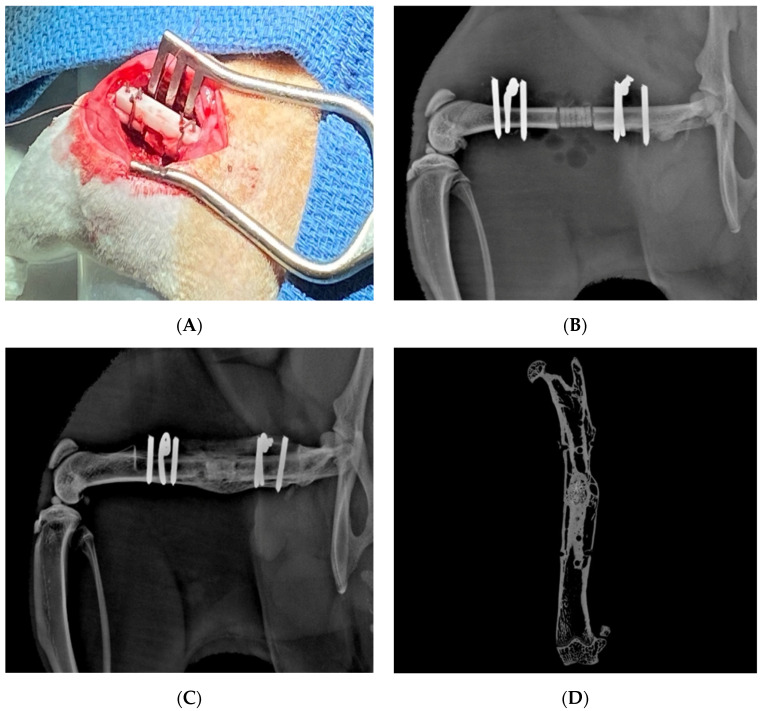
3D printed hydroxyapatite/tricalcium phosphate scaffold loaded with LV-TSTA-BMP-2 transduced rat bone marrow stem cells. (**A**) Intra-operative implantation of the scaffold into a 6 mm femoral defect in a Lewis rat. (**B**) Lateral X-ray image of implanted scaffold on postoperative day 0. (**C**) Lateral X-ray and (**D**) MicroCT images taken at 24 weeks demonstrating healing of the defect and incorporation of the scaffold.

Kang et al. modeled mandibular bone defects in vitro using a human amniotic fluid–derived stem cell-laden scaffold made from a combination of polycaprolactone and tricalcium phosphate and demonstrated 91% cell viability at 1 day after fabrication. The authors also investigated the same cell-laden scaffold in rat calvarial defects, and found that 5 months after implantation, there was a significant degree of angiogenesis and formation of both mature bone and osteoid, indicating an active remodeling process [120]. Park et al. used pneumatic microextrusion to create a polycaprolactone scaffold supplemented with BMP-2 laden collagen hydrogel and alginate–gelatin mixture. In vitro cell viability 1 day after scaffold fabrication was >90% with microvessel formation noted within the scaffold, and the volume of bone formation was found to be greater in scaffolds that had been pretreated with VEGF [127]. Zhai et al. reported successful treatment of rat tibial defects using a microextrusion scaffold with a hybrid bioink of N-acryloyl glycinamide and nanoclay seeded with rat osteoblast cells [128]. Similarly, Kang et al. demonstrated successful radiographic healing of critical sized rat femoral defects with extrusion printed Hyperelastic Bone^®^ (Dimension Inx LLC, Chicago, IL, USA) seeded with rat bone marrow cells transduced with a lentiviral vector containing the cDNA for BMP-2 (Figure 6) [129].

##### Laser-Assisted Bioprinting

Laser-assisted bioprinting (LAB) involves a laser source directed onto a disk containing an energy-absorbing ribbon and bioink, which is formed into droplets when pressure is generated from vaporization (Figure 5) [75]. As this technique is nozzle-free, it avoids cell clogging and exposure of cells to the mechanical stresses experienced in microextrusion printing while still maintaining high resolution of the printed constructs (Table 2) [114]. In addition to reports of high cell viability immediately after printing [122,123,124,125], in vivo assessments of LAB-generated scaffolds used for rat calvarial defects show robust bone regeneration and integration into the defect borders [130,131]. Keriquel et al. investigated the direct deposition of cell-free LAB-generated nano-hydroxyapatite slurry into rat calvarial defects and noted enhanced bone formation with no measurable damage to the dura when compared with laser-free controls [132]. The authors later employed an in situ approach where MSCs were printed onto a nano-hydroxyapatite/collagen disk and implanted into rat calvarial defects with substantial bone formation [130]. Current literature suggests that LAB may be a viable option for bone tissue engineering, but its applicability is still limited by the relatively high production cost as compared to other 3D printing technologies and the time-consuming nature of the process [114].

#### 3.2.6. Electron Beam Melting

Electron beam melting (EBM) is a frequently used 3D printing technique for the generation of metallic implants. It is a subtype of powder bed fusion-based additive manufacturing that uses an electron beam to sequentially melt thin layers of a starting material with conductive properties, such as metallic alloys [133,134]. EBM has particular utility in the generation of porous metal scaffolds due to the high efficiency and resolution provided by the electron beam, in addition to preservation of mechanical properties [135,136]. Odegaard et al. cultured bone-marrow derived stem cells on EBM-generated titanium-aluminum alloy (Ti6Al4V) scaffold and demonstrated robust osteogenic differentiation, cell viability, and proliferation within the scaffold [137].

Tantalum is a porous metal that has been used to create EBM-generated scaffolds **to** treat bone defects [133,138]. Wauthle et al. implanted an EBM-generated porous tantalum scaffold into 6 mm rat femoral defects and found that new bone formed both around and within the pores of the scaffolds, resulting in near-bridging of the defect [139]. However, the explanted bones underperformed during biomechanical testing when compared to controls, indicating that augmentation with cells, growth factors, or gene therapy may be necessary when adapting this strategy for humans.

Commercially, EBM is frequently utilized to fabricate highly porous metal implants, typically Ti6Al4V, for a broad range of orthopedic applications to allow for osseous ingrowth [140]. Hou et al. combined an EBM-generated porous titanium prosthesis with an intramedullary nail in five patients with irregular segmental femoral defects. None of the patients suffered from implant-related complications and the cohort reported good functional outcomes, while X-rays showed good bony interlocking at the interface of the implant with native bone [141]. Customized EBM-generated tantalum scaffolds have also been used in the context of human hip, fibula and femur defects with satisfactory functional results [138]. A potential disadvantage of this strategy is that the implants are used to replace lost bone rather than creating an environment that allows for bone regeneration and remodeling.

#### 3.2.7. Other Additive Manufacturing Techniques

Though employed less frequently, other additive manufacturing techniques have been used in bone tissue engineering contexts. Selective laser melting (SLM) is similar to EBM but uses a high-power laser instead of an electron beam. It allows for a broader range of starting materials, including metallic alloys, biodegradable metals such as magnesium and zinc alloys, and ceramics such as tricalcium phosphate and glass [142]. SLM affords a high degree of architectural complexity that can mimic native bone, and titanium scaffolds specifically have demonstrated excellent biocompatibility, mechanical strength, and osseointegration [143,144,145,146]. Clinical applications have centered around dental prostheses [147,148], though the successful implantation of an SLM-generated titanium alloy bone plate into a patient who suffered a pelvic fracture shows promise for future applications in load-bearing bones [149].

Another interesting additive manufacturing techniques is directed energy deposition (DED), which consists of the delivery of a heated substrate, typically a metallic alloy, to the nozzle head, where a high-energy laser is simultaneously focused, to generate a layered construct in a melt pool [150]. DED is unique in that multi-material constructs can be rapidly fabricated, though its use in a biomedical context has been limited due to difficulty in controlling the resolution of the generated construct, especially surface topography [151,152]. However, DED may have some clinical utility when used to coat metallic implants intended for load-bearing applications, as it has been shown to improve in vitro cell viability, cell-scaffold interactions, and bony ingrowth while retaining mechanical strength when compared to non-coated implants [153,154,155]. Since DED has not been investigated in models, its potential utility to treat segmental bone defects is unknown.

### 3.3. 3D-Printing Scaffold Materials 

**Figure 7:**
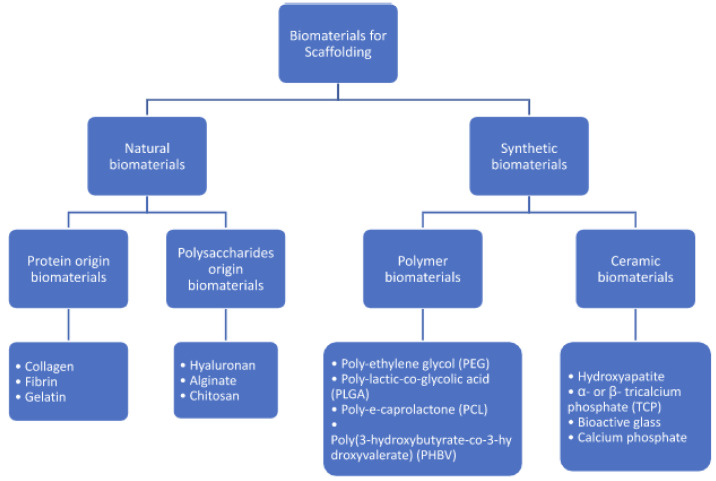
Biomaterials for 3D-printed Scaffolds. Adapted from: Alaribe, F. N. et al., 2016 [156].

An overview of the biomaterials commonly used for 3D-printed scaffolds is shown in Figure 7.

#### 3.3.1. Hydroxyapatite 

Hydroxyapatite (HA) is a mainstay in the production of scaffolds for bone regeneration given its osteoconductivity, bioactivity, and bioaffinity [157]. Furthermore, as it is composed of only calcium and phosphate, it has very low potential for local or systemic toxicity or immunoreactivity. Unlike the traditional methods of hydroxyapatite scaffold production, the use of 3D printing methods confers the ability to control the geometry of the scaffold, as well as its pore size and shape, allowing for the construction of customizable implants with complex and unique architecture [158]. The utility of 3D-printed hydroxyapatite scaffolds has been demonstrated in a number of in vitro and, importantly, preclinical in vivo studies. For example, 3D-printed Hyperelastic Bone^®^, a commonly used hydroxyapatite composed of 90% HA, has shown promise in its use in critical-sized mandibular defects, wherein it was found to upregulate production of type 1 collagen and VEGF, as well as promote de novo osteogenesis and tissue mineralization [58]. Chakraborty et al. demonstrated substantial bone in-growth within the Hyperelastic Bone^®^ scaffold when implanted into 3 mm rat tibial defects [159].

Incorporation of 3D-printed hydroxyapatite with other materials has also shown promise in addressing one of its main fallbacks: its slow resorption rate [157]. Biphasic calcium is one such mixture composed of HA and β-tricalcium phosphate and has shown promise in vivo. Kim et al. reported significantly improved bone formation in critical-sized mandibular dog defects treated with 3D-printed biphasic calcium scaffolds compared to untreated negative controls [160]. 

#### 3.3.2. Calcium Phosphates 

Calcium phosphates are the most commonly utilized ceramic in 3D-printed bone scaffolds. Of these, tricalcium phosphates (TCP), particularly β-TCP, are highly ubiquitous. As with hydroxyapatite, the use of 3D printing has allowed for finer control of their architecture, porosity, and geometry. The methods used in the 3D printing of TCP include SLA, robocasting and sintering, the latter of which has the benefit of temperature control for the optimization of the scaffold’s mechanical properties [161]. The promise of tricalcium phosphates lie in their biocompatibility, osteoinductivity, and favorable mechanical properties, all of which have been demonstrated in both in vitro and in vivo studies [162,163,164]. Tover et al. fabricated a 100% β-TCP scaffold implanted into critical sized radial defects in rabbits and found evidence of new bone formation and ingrowth with biomechanical parameters similar to native bone, and no evidence of immunoreactivity [162]. More recently, a 3D-printed TCP scaffold supplemented with the osteogenic factor dipyridamole was used in the reconstruction of rabbit calvaria and alveolar defects. Histological analysis demonstrated evidence of organized and vascularized bone that possessed mechanical properties similar to that of native bone [163]. 

As with hydroxyapatite, the incorporation of tricalcium phosphate with other scaffold materials has been extensively investigated [165]. In vitro work by Park et al. demonstrated the effects of a β-TCP/polycaprolactone (PCL) scaffold on human adipose and bone marrow derived stem cells, in which the scaffolds promoted significantly higher osteogenic differentiation, gene expression, and levels of ossification proteins compared to negative controls [166]. Several animal and human case reports also highlight the potential use of 3D-printed tricalcium phosphate alone or supplemented with recombinant BMP-2 in bone defect models [167,168]. Findings indicate favorable outcomes, with almost complete functional recovery in a canine model of radial atrophic union after 36 months [168], and appreciable production of new bone and complete scaffold integration 7 years after implantation into a maxillary alveolar ridge defect in a human patient [169]. 

#### 3.3.3. Bioactive Glasses 

Bioactive glass (BG) has emerged as a promising alternative material for 3D-printed scaffolds due to its demonstrated osteoconductivity and biocompatibility, and it is widely utilized as both a drug-delivery system and as a component of implants [170,171]. BG can be used as a raw material for a variety of 3D printing techniques, including SLS, SLA, and FDM [171]. Tulyaganov et al. demonstrated the effectiveness of 3D-printed silica-based BG in forming hydroxyapatite within simulated body fluid (SBF), as well as an in vivo 4.5 mm rabbit femoral defect model, in which the 3D-printed BG scaffolds resulted in osseous defect healing and the formation of new bone [172].

Mesoporous bioactive glass (MBG) contains a highly interconnected large-pore network similar to that of subchondral bone and has been examined as an additive to a variety of different scaffold materials to further promote bone repair [173,174]. Zhang et al. observed that the addition of MBG to 3D-printed β-TCP scaffolds promoted significantly enhanced compressive strength and mineralization, as well as cell attachment, viability, and angiogenic gene expression of human umbilical vein endothelial cells (HUVECs) when compared to β-TCP scaffolds alone. In vivo, the composite scaffolds also resulted in significantly more new bone formation within rabbit calvarial defects [173]. Similarly, Pant et al. demonstrated that a 3D-printed polylactic acid (PLA)-MBG composite scaffold exhibited superior biomechanical properties and in vitro cell attachment and proliferation, mineralization, and gene expression when compared to PLA alone [174]. Despite its observed efficacy, bioactive glass is structurally brittle when used alone, which can limit its utility as the optimal choice for repair of load-bearing bones, and often necessitates their combination with other scaffold materials [175].

#### 3.3.4. Polymer Addition 

The addition of various polymers to hydroxyapatite and tricalcium phosphate 3D-printed scaffolds can help address some limitations, such as structural brittleness and unfavorable mechanical properties [176]. Natural hydrogel polymers such as alginate, hyaluronic acid, and chitosan, when used as cell-laden bio-inks in additive manufacturing, can improve biocompatibility, biodegradability and provide an environment that promotes cell adhesion and proliferation [177]. Ye et al. added a chitosan (CS) coating to a 3D-printed poly(3-hydroxybutyrate-co-3-hydroxyvalerate) scaffold and found that compared to non-coated scaffolds, CS coating resulted in improved adhesion and proliferation of rat bone marrow stromal cells and upregulation of osteogenic genes [176]. 

Synthetic biodegradable polymers have also been investigated in bone tissue engineering owing to demonstrated biocompatibility, biodegradability, and mechanical strength [178]. Poly (lactic-co-glycolic acid) (PLGA) is a commonly used synthetic polymer in bone tissue engineering, owing to its biocompatibility and minimal toxicity [179]. The use of PLGA as a nanocarrier of TGF- β1 in 3D-printed collagen-based scaffolds resulted in growth factor release that was comparable to that seen in the extracellular matrix (ECM) of native bone [180]. Bioactive glass, limited in its use alone by mechanical brittleness, is often a substrate for polymer additions [175]. A PLA-BG scaffold was found to have favorable biomechanical properties such as elastic modulus and compressive strength, while a combination of bioactive glass and poly(propylene fumarate) fabricated by Kleinfehn et al. resulted in a functionalized scaffold through catechol molecule tethering and increased bioglass concentrations [74,181]. Combining the polymer polycaprolactone (PCL) with β-TCP in the complex reconstruction of zygomatic-maxillary defects, CT assessment of the volume and density of bone formation indicated a satisfactory ratio of new bone formation to implant volume [182]. Furthermore, there is interest in the use of self-healing elastomers, which rely on the use of photo elastomer inks containing both thiol and disulfide groups to interact for photocuring capacity [183]. These findings underscore a promising utility of polymers in 3D-printed scaffolds to treat segmental bone defects.

#### 3.3.5. Metals

A variety of metallic materials have been investigated as components of scaffolds for bone tissue engineering due to favorable characteristics, including compressive strength suitable for load-bearing applications, as well as corrosion resistance. However, their use has been met with concerns, related to the potential for cytotoxicity and biodegradability. Titanium alloys are commonly used components of 3D-printed scaffolds applied to bone defects. Xu et al. utilized additive manufacturing to fabricate a porous titanium-based scaffold that aimed to address the issue of aseptic implant loosening by achieving an elastic modulus similar to that of native bone. In vivo implantation into rat 6 mm femoral defects demonstrated excellent new bone formation after 8 weeks, as well favorable osseointegration and hemocompatibility of the scaffolds with surrounding tissue [184]. 3D-printed titanium implants have been used clinically in the skull defects of 21 patients between 2013 and 2015 [185]. 3D computed tomography was utilized in conjunction with electron beam melting to fabricate constructs with a precise fit; with final follow-up demonstrating satisfactory fixation of the implants, with complications reported in only one patient [185]. This series highlights a potential clinical application for metal-based 3D-printed constructs for bone replacement.

Certain biodegradable materials such as iron (Fe), magnesium (Mg), and zinc (Zn) are of particular interest in bone tissue engineering, owing to their gradual corrosion in vivo, thereby improving their osseointegration with native bone. Yang et al. utilized an iron-based scaffold that demonstrated a compressive strength similar to that of natural bone. When combined with HA nanoparticles, the composite scaffold promoted increased alkaline phosphatase production and osteogenic differentiation of rat bone marrow mesenchymal stem cells (rBMSCs), underscoring a synergy between the scaffold’s components [186]. As a component of native bone, the biocompatibility and osteoinductivity of magnesium make it an attractive material to incorporate as part of 3D-printed scaffolds. Compared to pure TCP scaffolds, composites containing higher concentrations of magnesium displayed enhanced Alizarin Red Staining, ALP activity, osteogenic gene expression, and bone formation when implanted into a rabbit femoral condyle model [187]. A zinc-hydroxyapatite composite made by a sintering process showed a favorable corrosion profile [186]. In vitro testing with pre-osteoblastic cells demonstrated cell viability within the scaffold, and an in vivo rat femoral condyle model showed excellent new bone formation over time [186].

### 3.4. 3D-Printed Scaffold Architecture 

#### 3.4.1. Pore Size

Pore size plays a crucial role in promoting the attachment of cells onto the scaffold, as well as their migration into and proliferation within the construct. One of the most important benefits of 3D printing techniques is that they allow for precise control over architectural details, and therefore the determination of an optimal pore size or range is critical in the development of scaffolds of increasing scale and complexity [93,188]. Lim et al. evaluated the effect of pore size on the in vivo efficacy of biphasic calcium scaffolds composed of HA and β-TCP in rabbit calvarial defects and found that larger pore sizes (1200–1400 µm) were associated with enhanced bone formation, although this effect disappeared at 8 weeks [189]. However, Entezari et al. found a minimum pore size of about 400 µm was ideal for new bone formation, with an upper limit of approximately 600 µm [190].

Zhang et al. assessed the effect of different pore sizes (400, 700, and 900 µm) in porous titanium scaffolds fabricated via selective laser melting. An in vitro study using rat bone marrow stem cells seeded on the scaffolds demonstrated that a pore size of 600–700 µm with a porosity of 70% had enhanced cell viability, expression of osteogenic-related genes, and ALP activity. In a rat femoral defect model, a pore size of 900 µm was found to promote the highest level of bone volume to tissue volume (BV/TV) on microCT [191]. In a rabbit femoral defect model, a SLM-generated titanium implant with a pore size of 600 µm was associated with superior bony ingrowth and mechanical properties compared to 200 µm and 900 µm [192]. Although the majority of the literature on this topic suggests that a pore size in the range of 400 µm to 600 µm is ideal for 3D-printed bone tissue engineering, smaller and larger pores have been found to also be adequate [189,190,191,193]. In addition, pores of the same size in scaffold made via different 3D printing techniques or different starting materials may possess different biomechanical osteogenic properties [194]. This lack of consistency highlights the continued need to characterize the effect of pore size and porosity in different biologic environments.

#### 3.4.2. Pore Geometry and Patterning 

The ability conferred by 3D printing techniques to precisely control the architecture of scaffolds for bone tissue engineering has allowed for more complex customization of the shape and geometry of pores, as well as their patterning and distribution throughout scaffolds. The association between pore geometry and mechanical properties was investigated by Zhang et al., in which different angles of layer overlap were used to create various pore geometries, which resulted in changes in Young’s moduli and compressive strengths as the scaffolds were compressed along different planes [195]. Ferlin et al. conducted an in vitro comparison between cubic and cylindrical pore geometries and found that the cubic geometry resulted in significantly higher numbers of mesenchymal stem cells (MSCs) undergoing adipogenesis and chondrogenesis [196]. Interestingly, the cylindrical shape was associated with increased expression of osteogenic markers at earlier time points, whereas the cubic pore shape proved superior at later ones [196]. Several in vivo studies assessing various pore geometries have found relatively similar degrees of bone formation within defects, though Kolan et al. reported more fibrous tissue formation with their diamond shaped BG scaffold [197,198].

Patterns of pore distribution within 3D-printed scaffolds can also impact bone regenerative capacity and mechanical properties. Greater irregularity of pore distribution was found to be associated with improved resistance to compressive forces in a rigorous comparison of different pore sizes and regular pore distribution [199]. Hallman et al. set out to delineate the effect of pore alignment on the in vivo bone growth of a 3D-printed HA/DMB scaffold in a rat spinal fusion model. The highest fusion rates and osseointegration were seen with 1000 µm pores in which the struts were aligned. Further, in comparison to non-porous scaffolds, full vascularization was seen within porous constructs [193]. Wang et al. fabricated PCL scaffolds with an upper pore size of 200 µm and lower pore size of 400 µm to promote cartilage and bone regeneration, respectively. The scaffolds were found to facilitate the migration of BMSCs around and within the scaffold, as well as their differentiation along osteogenic and chondrogenic pathways [200]. As with pore size, the lack of a consistently demonstrated benefit with one type of pore geometry or patterning underscores the need for continued studies.

#### 3.4.3. Surface Topography 

The surfaces and internal spaces of 3D-printed scaffolds play distinct roles in bone tissue engineering. Namely, scaffold surfaces have been implicated in cell adhesion, while the internal structure should allow for appropriation migration of cells into the scaffolds [201]. Numerous surface characteristics influence cellular adhesion and implant integration including surface patterning, porosity, and roughness [202]. These characteristics can be modified in different ways including acid etching, sandblasting, electrospinning and polydemixing. Zamani et al. created 3D-printed PCL scaffolds that were modified with NaOH to create a honeycomb-like surface pattern and seeded them with pre-osteoblasts. Compared to untreated controls, in vitro assays of the modified scaffolds displayed increased collagenous matrix deposition and enhanced mineralization [203]. Vu et al. demonstrated that compressive moduli were similar between 3D-printed TCP scaffolds regardless of surface topography. However, the ridge-pattern scaffolds had the highest Young’s (elastic) modulus, and all surface-modified scaffolds exhibited improved osteoblast proliferation compared to the unmodified controls [204].

### 3.5. 3D-Printed Scaffold Augmentation 

#### 3.5.1. Platelet Rich Plasma 

Platelet-rich plasma (PRP) is a concentrate isolated from whole blood subjected to centrifugation with the ability to improve the hydrophilicity and biocompatibility of scaffolds used for bone tissue engineering [205]. Much of the utility of PRP lies in its ability to promote the release of cytokines such as VEGF, IGF-1, and TGF-β from scaffolds. Liu et al. demonstrated that a 3D-printed PCL/β-TCP scaffold combined with PRP/gelatin microspheres successfully sustained release of cytokines over a period of three weeks, which promoted increased survival, proliferation, and migration of bone marrow mesenchymal stem cells compared to non-augmented scaffolds [206]. Despite this demonstrated benefit of PRP, its storage has proven to be a challenge. Li et al. explored a novel freeze-drying method to circumvent this issue using PCL scaffolds augmented with freeze-dried PRP compared to traditional PRP and found increased expression of osteogenic genes including ALP, osteocalcin, and osteopontin within the freeze-dried group [207]. The augmentation of biomimetic scaffolds with PRP is a promising avenue that has been shown to have an appreciable effect on the osteogenic ability of bone repair scaffolds. However, it is not clear how this strategy would be adopted for clinical use since the patients’ blood would have to be drawn and the PRP would then be applied to the scaffold.

#### 3.5.2. Stem Cells

The augmentation of scaffolds used for segmental bone defect repair with stem cells have reproducibly resulted in enhanced osteogenic potential, with expansion to 3D-printed scaffolds in recent years [126,208,209,210]. Mesenchymal stem cells derived from the bone marrow (BMSCs) are commonly used in bone tissue engineering as they are able to effectively differentiate into a variety of cell types including osteoblasts and chondroblasts [211]. Chi et al. demonstrated that BMSC-seeded HA scaffolds exhibited increased ALP activity and expression of osteogenic genes compared to an HA-only scaffold, and this was mirrored by an in vivo analysis in which addition of BMSC-ECM resulted in enhanced bone repair [209].

Adipose-derived stem cells (ASCs) have been used increasingly in lieu of BM-derived stem cells in pre-clinical bone tissue engineering studies due to challenges in expansion of BMSCs and the associated donor site morbidity [212]. Kurzyk et al. reported excellent ASC proliferation in culture as well as high efficiency with scaffold seeding, which improved with increased volumes of cell suspension and propagation time [210]. Alternatives to BMSCs and ASCs have been explored in an effort to eschew invasive human stem cell isolation methods. Hashemi et al. assessed the osteogenic differentiation potential of human induced pluripotent stem cells (hi-PSCs) seeded onto 3D-printed collagen-coated biphasic calcium scaffolds. When compared to buccal fat pad stem cells, hi-PSCs resulted in increased upregulation of osteogenic genes in vitro and improved in vivo bone formation and expression of collagen I and osteocalcin in rat calvarial defects [126]. Due to the demonstrated superior osteoinductivity of 3D-printed scaffolds augmented with stem cells, continued investigations into optimal sources and scaffold-seeding techniques have the potential for clinical adaption, but further study is necessary in clinically relevant animal models. 

#### 3.5.3. Antimicrobials 

Infections of bone and joint spaces in the setting of concomitant bone loss pose a unique and recurrent challenge in orthopedics, resulting in exploration of 3D-printed scaffolds augmented with antimicrobial compounds. This approach involves either the incorporation of specific antibiotics into the scaffolds or the addition of materials with demonstrated antimicrobial effect. Qiao et al. combined a PLLA-based 3D-printed scaffold with PLGA microspheres containing rifampicin and moxifloxacin. In vitro studies confirmed the cytocompatibility of this composite, and an in vivo model of an infected rabbit bone defect showed superior anti-infection and bone defect repair ability compared to PLLA alone [213]. Marsh et al. examined an antimicrobial augmentation of their 3D-printed silicate bioactive glass (BG) scaffold, in this case by the addition of antibacterial silver (Ag) to form an Ag-BG ceramic. Inoculation of the scaffolds with methicillin-resistant S. aureus (MRSA) resulted in a significant decrease in the viability of the bacteria, underscoring a potential effect against the persistently challenging pathogen [214]. This is a challenging clinical model because the impact of the kinetics of the release of these compounds must be determined as well as the material requirements for scaffold incorporation into the bone.

#### 3.5.4. Growth Factors 

##### Bone Morphogenic Protein (BMP)

The effect of BMPs on the bone growth and healing process have been very well described [27,28]. This utility has resulted in the frequent use of BMP proteins in bone tissue engineering, and the advent of 3D printing techniques portends an exciting opportunity for their expanded application. Bae et al. described the use of a 3D-printed scaffold composed of PCL/β-TCP/bdECM (bone decellularized extracellular matrix) that was conjugated with rhBMP-2 in an in vivo study of mandibular defects. The rhBMP-2 augmented scaffolds displayed a significantly higher bone-to-implant ratio, although new bone volume percentage was similar to non-augmented controls [215]. Cao et al. utilized a primate mandibular defect model to assess the effects of rhBMP-2 coating on their PLGA/TCP scaffold. Although in vitro cytoskeletal and nuclear staining of co-cultured hBMSCs found no significant difference between the two groups in cell proliferation and differentiation, the in vivo microCT analysis of bone volume/total volume revealed a significantly increased amount in the augmented group [216]. Lauer et al. investigated 3D-printed porous PLA cylinders functionalized with either BMP-7 or stromal derived factor-1 (SDF-1) in critical-sized femoral rat defects, and found that scaffolds with BMP-7 conferred the most distinct effect on new bone formation, even compared to SDF-1. However, the use of BMP-7 also resulted in heterotopic ossification [217].

The optimal loading and delivery method of BMP within 3D-printed scaffolds is an area of continued interest. Huang et al. utilized a mesoporous calcium silicate (termed “mesoCS”) 3D-printed scaffold to evaluate two different BMP-2 loading methods: pre-loading BMP-2 with mesoCS prior to 3D printing, or direct-loading once the scaffolds have been printed. Although the directly loaded scaffold released 50% more BMP-2 than the pre-loaded scaffold initially, the latter ultimately maintained a more sustained release profile over two weeks [218]. More recently, a novel core/shell microsphere (CSM) delivery construct was developed by Zhuang et al. in an attempt to promote sustained BMP-2 release. This consisted of a heparin-coated PLA microsphere core surrounded by alginate shells, which were evaluated in vitro and then placed into rat segmental defects. Compared to alginate-only microspheres (AM), BMP-2 release from the CSM was lower initially but sustained a longer-lasting release profile. The CSM-BMP-2 resulted in increased osteogenic gene expression and an increased BV/TV ratio when compared to AM-BMP-2 and negative controls [219]. 

These studies underscore the promise of BMP, particularly BMP-2, as an adjunct to 3D-printed scaffolds in bone tissue engineering. Further work to optimize loading and delivery, as well as understanding the influence of 3D-printed scaffold resorption time needs to be better characterized to fully realize the potential of this scaffold augment and its clinical applications. 

##### FGF

Fibroblast growth factor (FGF) is a mitogen that promotes cell proliferation and differentiation, especially in the contexts of angiogenesis and osteogenesis. Its osteogenic effect is belied by an increased cellular content of osteocalcin and number of osteoblasts, more prominently seen with the basic subtype of FGF (bFGF). The effect of bFGF on inducing osteogenic differentiation and cellular proliferation of cultured stem cells has been well-studied for the past two decades, with most suggesting that it promotes these processes [220].

More recently, the role of FGF in promoting bone healing has been investigated in 3D-printed scaffolds. Lin et al. printed a graphene-containing silicate/PCL scaffold augmented with FGF that was cultured with Wharton’s Jelly derived mesenchymal stem cells (WJMSCs). Compared to an FGF-R knockout (KO) group, cell proliferation, ALP activity, and expression of osteogenic (ALP and osteocalcin) and angiogenic (VEGF and vWF) proteins were significantly increased [221]. Lai et al. carried out a similar study using a 3D-printed calcium silicate scaffold. Gelatin and magnesium were added in order to improve growth factor loading onto the scaffold and genipin cross-linking helped to regulate scaffold degradation [222]. In comparison to a control group not loaded with FGF, WJMSCs exhibited increased biocompatibility, ALP activity, and osteogenic marker expression when cultured with the experimental scaffold. In vivo, FGF was found to promote significantly elevated trabecular thickness and bone volume/tissue volume by microCT at 8 and 12 weeks within a rabbit femoral defect [222]. Overall, these studies highlight a promising role for FGF in conjunction with 3D-printed scaffolds for segmental bone defects. However, studies in more rigorous animal models are needed to characterize the potential of FGF more clearly in 3D-printed bone regeneration. 

##### VEGF

The presence of a reliable blood supply is a crucial element of successful bone healing to ensure the delivery of oxygen, nutrients, growth factors, and circulating cell types [223]. Vascular endothelial growth factor (VEGF) is a well-known driver of angiogenesis across a variety of different cellular and physiological processes and has been investigated as an addition to bone tissue engineering scaffolds to promote vascular ingrowth. Sharmin et al. demonstrated the utility of an allograft augmented with VEGF and BMP in promoting new bone formation in segmental rat femoral defects when compared with non-functionalized control grafts. The in vitro analysis demonstrated an increased amount of TRAP+ osteoclasts in bone marrow derived stem cells co-cultured with the released VEGF, which may point towards a role in scaffold resorption and remodeling [224,225]. 

As additive manufacturing techniques have become more readily available, the augmentation of 3D-printed scaffolds with VEGF has also been investigated. Fahimipour et al. investigated a 3D-printed VEGF-loaded gelatin/alginate/B-TCP composite scaffold seeded with human umbilical vein endothelial cells and found increased cell numbers and osteoblast viability, adhesion, and proliferation on the scaffold [226]. In a rat calvarial defect model, Chakka et al. utilized PLA-based 3D-printed scaffolds coated with polydopamine and VEGF-coding DNA plasmid and observed a 1.6-fold increase in new bone formation compared to negative controls and increased neo-angiogenesis within the newly formed bone [227]. The optimal delivery and release kinematics of VEGF-coated scaffolds were evaluated by Chen et al. who found that addition of calcium sulfate (CS) to hydroxyapatite scaffolds resulted in increased and stable VEGF release [228].

In addition to VEGF, deferoxamine (DFO) been investigated for its ability to promote vascularization of 3D-printed constructs. DFO has been shown to facilitate vascularization and bone production by promoting the expression of pro-angiogenic genes [229]. A DFO augmented PCL-based scaffold demonstrated accelerated vascularization, increased production of mineralized matrix and osteogenic differentiation of in vitro mesenchymal stem cells. There remains a need to evaluate the use of VEGF with different 3D-printed materials, as well as in conjunction with different growth factors including those previously described.

#### 3.5.5. Gene Therapy

Gene therapy broadly refers to the practice of delivering genes to a site of interest or otherwise modifying them by silencing, inactivation, or replacement [230]. The use of gene therapy in bone tissue engineering carries potential for segmental defect healing. The challenges associated with in vivo gene therapy include the possibility of systemic toxicity linked to direct inoculation of a host with a viral vector and a lack of responding cells in stringent biological environments associated with segmental bone defects. Therefore, there is interest in developing ex vivo gene therapy because the cellular delivery vehicle can be selected depending on the clinical circumstance. This technology involves extracorporeal delivery of genes of interest to target cells, typically stem cells, and subsequent implantation into the host [211]. The utility of this technique in bone healing has been demonstrated in several studies that have primarily utilized genes encoding BMP-2 within either adenovirus (AD) [231,232,233,234,235] or lentivirus (LV) vectors [235,236,237,238,239]. Robust radiographic healing of bone defects using ex vivo regional gene therapy has been demonstrated using rat bone marrow stem cells transfected with AD-BMP-2 [231]. Another ex vivo application demonstrated a greater than 90% union rate in nude rat femoral defects treated with human ASCs transduced with a LV-BMP-2 vector [239].

The use of 3D-printed scaffolds provides a novel method of effectively delivering transduced cells as a part of a gene therapy strategy to heal segmental bone defects. A 3D-printed composite scaffold containing PCL/PLGA and HA nanoparticles was used to deliver miR-148b, a micro-RNA fragment implicated in osteogenesis, directly to rat bone marrow cells and rat critical sized calvarial defects. In vitro, the bone marrow cells underwent early differentiation and expressed osteogenic markers as confirmed by an immunohistochemical assay, while microCT and histology showed improved bone regeneration to the point of near-complete repair of the in vivo model [240]. Alluri et al. utilized 3D-printed Hyperelastic Bone seeded with LV-transduced human ASCs overexpressing BMP-2 implanted in rat hind limb muscle pouches and found significant increases in osteogenesis [241]. Further studies investigating 3D-printed TCP scaffolds augmented with LV-transduced rat BMSCs in critical sized femoral defects demonstrated complete radiographic healing compared to controls [164]. This finding was reproduced using 3D-printed Hyperelastic Bone seeded with LV-transfected rat BMSC in a rat femoral defect model (Figure 6) [129]. In both cases, the augmented scaffolds promoted significant increases in new bone formation seen on microCT and histomorphometry [129,241]. The results of these studies demonstrate the potential of 3D-printed scaffolds to be used as a cellular delivery vehicle for transduced cells. The challenge now is to test the efficacy of this strategy in large animals to see if larger scaffolds can be constructed to effectively deliver genetically manipulated mesenchymal stem cells to a large bone defect.

## 4. Conclusions and Future Perspectives

In conclusion, 3D printing represents a promising technology in the management of segmental bone loss in orthopaedics surgery that offers several benefits compared to traditional techniques. First, 3D printing avoids the donor site morbidity and risks of autograft harvest as well as the risk of disease transmission and host immune response associated with allograft. Second, the use of 3D printing may allow for optimization of the mechanical and osteoinductive properties of the scaffold through modulation of material composition, scaffold geometry, and topography that can be tailored to specific bone defect locations. Furthermore, the augmentation of scaffolds with growth factors, stem cells, or genetically manipulated cells (regional gene therapy) permits osteoconductive and osteogenic properties to enhance bone formation. Despite the significant advances in 3D-printed scaffolds, it is essential that these scaffolds be evaluated in clinically relevant animal models to demonstrate that not only large scaffolds can be constructed, but these scaffolds can be incorporated into host bone when seeded with cells or growth factors. While 3D-printed metal-based bone replacement strategies exist within orthopedic oncology and revision joint arthroplasty, to our knowledge, 3D-printed constructs with growth factors to promote bone regeneration are not available in clinical practice. Throughout this review we highlight the emerging topics and techniques for 3D printing in regeneration of critical sized defects. With more thorough tests in the future, the strategy of using 3D-printed scaffolds customized for each patient has the potential to revolutionize the treatment of large bone defects in humans.

## Figures and Tables

**Table 1:** Summary of current 3D printing techniques.

Technique	Description	Advantages	Disadvantages	Literature
Fused Deposition Modeling (FDM)	Extrusion of plastic polymers from a heated nozzle onto a cooler substrate, allowing for rapid solidification Resolution: Low	Reliable and accurate Fast and inexpensive process	High temperatures require cooldown period before scaffold use Molten phase restricts material use Secondary support necessary	[73,74,75]
Stereolithography (SLA)	UV light-based method that involves layered curing of a photopolymer resin or a mixture of ceramic slurry Resolution: High	Fabricates precise, high-resolution structures Fast printing Can be used alongside cells, proteins, and growth factors	Limited material selections Compromises on build quality of constructs	[75,77,78,79,80]
Selective Laser Sintering (SLS)	Involves the use of a CO_2_ laser that sinters sequential layers of a powdered raw material to create a 3D construct Resolution: Medium	Fabrication of smaller scaffolds with precise specifications No supports are needed Post-processing not necessary	Lower density scaffolds Relatively limited starting materials	[76,81,82,83]
Robocasting	A High-viscosity slurry bioink is dispensed by the printer nozzle in a layered fashion to create a 3D structure Resolution: Low	Utilizes relatively low temperatures allows for the printing of bioactive materials Precise microarchitectural modulation	Secondary support necessary Slow printing speed	[75,84,85]

**Table 2:** Summary of current bioprinting techniques.

Technique	Description	Advantages	Disadvantages	Literature
Inkjet Based (thermal and piezoelectric)	Involves dripping a low-viscosity ink onto a substrate based on a computer program to create a 3D construct Resolution: High	Inexpensive and relatively fast High cell viability Easily implemented	Low viscosity technique limits stock of available starting materials	[76,84,113,114]
Ink Extrusion Based	Involves the use of pneumatic air pressure or mechanical systems to continuously disperse bioinks simultaneously Resolution: Low	Compatible with various bioinks Different bioinks can be used simultaneously during fabrication process, allowing for printing of complex scaffolds High cell viability	Potential for cell damage from exposure to large mechanical and shearing pressures Limited resolution of final constructs	[75,114,118]
Laser Based	Involves a laser source directed onto a disk containing an energy-absorbing ribbon and bioink. Resolution: High	Nozzle-free process limits clogging Less exposure of cells to mechanical and shearing stresses during fabrication High resolution constructs High cell viability	Expensive Fabrication process is time consuming	[75,113,122,123,124,125,126]

## Data Availability

No new data were created or analyzed in this study. Data sharing is not applicable to this article.
